# Mapping QTLs for cold tolerance at germination and the early seedling stage in rice (*Oryza sativa* L.)

**DOI:** 10.1080/13102818.2014.978539

**Published:** 2014-11-20

**Authors:** Aloka Lanka Ranawake, Oliver Escano Manangkil, Shinya Yoshida, Takashi Ishii, Naoki Mori, Chiharu Nakamura

**Affiliations:** ^a^ Department of Agricultural Biology, Faculty of Agriculture, University of Ruhuna, Mapalana, Sri Lanka; ^b^ Plant Breeding and Biotechnology Division, Philippine Rice Research Institute, Science City of Munoz, Nueva Ecija, Philippine; ^c^ Hyogo Institute of Agriculture, Forestry and Fishery, Kasai, Japan; ^d^ Department of Bioresource Science, Laboratory of Plant Breeding, Graduate School of Agricultural Science, Kobe University, Kobe, Japan; ^e^ Department of Agrobioscience, Laboratory of Plant Genetics, Graduate School of Agricultural Science, Kobe University, Kobe, Japan

**Keywords:** cold acclimation, cold tolerance, germination, growth stage specificity, quantitative trait loci (QTLs), recombinant inbred lines (RILs), rice (*Oryza sativa* L.)

## Abstract

Cold tolerance is an important breeding target in rice production. We studied quantitative trait loci (QTLs) controlling cold tolerance at germination (CTG) and early seedling (CTS) stages, using recombinant inbred lines derived from a *japonica* × *indica* cross. CTG was evaluated based on the percentage rate of germination at 15 °C for 12 days after pre-incubation of imbibed seeds at 20 °C for 2 days. For CTS, seven-day-old seedlings grown at 25 °C were subjected to two consecutive periods of three-day cold stress at 4 °C with an intervening eight-day recovery at 25 °C. CTS evaluation was according to an arbitrary five-point rating system at the fifth day of recovery after each stress period. No correlations were found between CTG and CTS, while a weak correlation was detected between CTS after the first and second stress. By the composite interval mapping, five QTLs for CTG explaining 5.7%–9.3% of the total phenotypic variance (PVE) and nine for CTS with PVE of 5.8%–35.6% were detected. Only one of these QTLs was common, indicating growth-stage specificity of cold tolerance. Four of the five QTLs after the second cold stress were different from the ones after the first cold stress. Analysis of variance test showed significant interactions between alleles at the QTL sites and the two stress conditions with respect to the mean CTS scores. A possible involvement of cold acclimation and usefulness of *japonica* germplasms in breeding for cold tolerance in *indica* rice was discussed.

## Introduction

The genetic potential of crops for yield has largely been hindered by abiotic stress factors in agriculture, resulting in an estimated average yield loss of more than 50% for most major crops.[1] Rice is the staple crop providing over 40% of the human food energy intake and our demand for rice was predicted to increase by 50% until 2030.[2] To meet this increasing demand for rice, development of cultivars with high yield potential and robust and durable resistance to both biotic and abiotic stresses is needed.[3] Since rice has a tropical origin, cold or low temperature stress is an important risk factor among a variety of abiotic stresses in rice production.[4,5] In the temperate as well as high-elevation tropical and subtropical rice-growing regions, it has long been known that cold stress adversely affects rice growth throughout its development from germination till harvest and causes significant yield loss due to poor and slow germination, non-vigorous and slow vegetative growth, high spikelet sterility, delayed and less heading and poor grain filling.[6,7] Hence, development of cultivars tolerant to cold stress has long been an important breeding target in rice production.[5,8]

Fast and uniform seed germination and vigourous seedling growth are essential for stable crop establishment. Germination rate and post-germination early seedling growth are the two major traits directly related to seedling vigour. Cold stress often impairs and delays germination and seedling growth of rice to cause poor stand establishment and non-uniform maturation, particularly in the regions where a system of direct seeding cultivation is practiced under low local temperatures.[9–11] Cold tolerance is known to be a complex trait associated with a number of biochemical and physiological events in plants.[12] Cold tolerance is under control by various biological mechanisms involving cold sensing, transcriptional regulation and post-transcriptional processing.[13,14] In earlier studies, several major genes such as *Cts1* [15] and *Cts2* [16] were reported to play a key role in cold tolerance at the seedling stage in rice. Moreover, considerable efforts have been directed towards detecting and mapping of quantitative trait loci (QTLs) because of the complexity and polygenic nature of cold tolerance. A volume of studies has been conducted using different mapping populations, marker systems and experimental bioassay conditions to detect and evaluate QTLs associated with cold tolerance at different developmental stages, including germination (CTG) and seedling stages (CTS). A large number of QTLs have been identified and evaluated for CTG and CTS, among which *qLTG3-1*,[17,18] *qCTS12*,[19] *qCTS4* [20] and *qCtss11* [21] have so far been fine mapped on the rice genome and/or cloned, although their functions still remain unknown.

In QTL analysis, developmental stage of the target traits is one important factor among others. In the tribe Triticeae, growth-stage specificity of cold and freezing tolerance is well documented.[22–24] In rice, both growth-stage specific and common QTLs have been reported for cold tolerance.[8] Andaya and Mackill [25,26], using the same set of recombinant inbred lines (RILs) derived from a cross of *japonica* M202 and *indica* IR24, reported the presence of stage-specific major QTLs for CTS on chromosomes 11 and 12 and others active at the booting stage, on chromosomes 2 and 3. With respect to CTG and CTS, the presence of stage-specific QTLs has been suggested based on studies using the same sets of mapping populations.[27–31] Severity of stress is another important factor in detecting QTLs for cold tolerance. For CTG, stress conditions at 10–25 °C have been applied, whereas lower temperatures ranging from 6 to 10 °C have often been used for CTS.[25,32–34] More severe conditions at constant temperatures at 4–5 °C have also been effectively used.[21,31,35]

We previously developed experimental protocols suitable for evaluating CTG and CTS, using a cold tolerant *japonica* cultivar ‘Hyogo-Kitanishiki’ (abbreviated as HGKN) and a cold sensitive *indica* cultivar ‘Hokuriku-142’.[36] Based on the protocols, we studied CTG and CTS of the RILs derived from this *japonica* × *indica* cross. In this study, we performed QTL analysis for CTG and CTS, using the previous bioassay data. For CTS, we also obtained new data after further subjecting the RILs to a second period of cold stress with an intervening recovery period after the first stress. We expected that the subjection of rice seedlings to the repeated and severer cold stress could help to detect not only a robust but also a different set of QTLs. Based on the results obtained, we discuss a possible involvement of a cold acclimation process for CTS in rice.

## Materials and methods

### Plant materials

A mapping population consisting of 162 RILs at F_6_ generation was derived from a cross between a cold tolerant *japonica* cultivar, HGKN, as the female parent, and a cold susceptible *indica* cultivar, ‘Hokuriku-142 or Yume-Toiro’ (HOK), as the male parent. HGKN is a sake-brewer's cultivar bred from a cross between two *japonica* cultivars, ‘Nada-Hikari’ and ‘Gohyaku-Mangoku’, in the Experimental Station of Sake-Brewing Rice, Hyogo Prefecture, Japan.[37] HOK was bred from a cross between an IRRI *indica* line ‘IR-2061-214-31’ and a Korean *japonica*–*indica* Tongil variety ‘Milyang 21’ in Hokuriku Agricultural Experimental Station, Japan.[38] HOK is a high-yielding cultivar with a high content of amylose and a low content of unsaturated fatty acids in the endosperm, both traits being considered desirable for sake brewing. Original objectives of this cross were to introgress simultaneously the favourable traits of HOK into HGKN and cold tolerance of HGKN into HOK. RILs were advanced according to the single seed descent method. The parental cultivars were used as controls.

### Bioassay conditions for evaluating CTG and CTS

Bioassay conditions for CTG and CTS after the first cold stress were according to our previous report.[36] Briefly, for CTG, seeds of HGKN and HOK were exposed to 50 °C for five days to break dormancy and surface sterilized by dipping in 70% (v/v) ethanol for 1 min and in 1% (w/v) solution of NaOCl for 1 h followed by washing three times in sterilized distilled water. Imbibed and surface-sterilized seeds were then placed in wet Petri dishes and incubated under continuous cold stress at 15 °C in dark after pre-incubation for two days at 20 °C. The pre-incubation was added as a modification from the method recommended by Sasaki [39], because both cultivars failed to germinate within a one-month period without the pre-incubation. After confirming a clear cultivar difference, 20 seeds from each of the parental cultivars and RILs were subjected to the developed bioassay conditions, and the whole experiment was repeated three times. The number of germinated seeds (scored based on plumule length, i.e. plumule longer than 5 mm) was recorded on the 12th day under these experimental conditions.

For CTS, germinating seeds in distilled water were planted in trays (24 × 24 cm) filled with soil. For each RIL, 20 germinating seeds were planted according to the randomized complete block design with four replicates each with five seedlings, and then grown for seven days under normal temperature at 25 °C with a photoperiod of 16 h light and 8 h dark. Seedlings were watered daily with 0.001% (w/v) Hyponex solution (N:P:K = 5:10:5 by volume, Hyponex, Japan). Cold stress was given twice in each experiment by subjecting seven-day-old seedlings to two consecutive periods of three-day cold stress at 4 °C with an intervening eight-day recovery period at 25 °C. At the end of the first cold stress, seedlings were returned back to 25 °C and the level of CTS was evaluated on the fifth day of the recovery period. The following arbitrary rating scale [36] was adopted: (1) the whole seedling became withered and died, (2) only the bottom part of the stem remained green, (3) the whole stem remained green, (4) the stem and one leaf remained green and (5) more than two leaves emerged and the stem and the leaves remained green. After the first CTS rating, the whole set of RILs were further subjected to a second period of three-day cold stress at 4 °C, and CTS was evaluated again on the fifth day of the recovery period at 25 °C, using the same arbitrary rating scale. The whole experiment was repeated three times.

### DNA extraction and marker genotyping

DNA was extracted according to the cetyltrimethylammonium bromide method.[40] Primer pairs for simple sequence repeats (SSR) from the International Rice Microsatellite Initiatives [41] were used in the polymorphism survey using DNA extracted from the parental cultivars. SSR markers were amplified in a 96-well plate, using the Gene Amp polymerase chain reaction (PCR) System 9700 (Applied Biosystems). PCR conditions were as follows: 5 min at 95 °C, 35 cycles of 95 °C for 1 min, 55 °C for 1 min and 72 °C for 2 min, with a final extension at 72 °C for 7 min. Marker genotyping of 162 RILs was according to Wu and Tanksley.[42]

### Construction of a linkage map and QTL detection

A total of 232 SSR makers showing clear band amplification were surveyed using the parental cultivars, among which 186 (80.2%) were polymorphic. A linkage map was constructed by MAPMAKER/EXP version 3.0b [43] based on 102 polymorphic SSR markers. Composite interval mapping was performed using Windows QTL CARTOGRAPHER version 2.5-011.[44] The logarithm of the odds (LOD) scores for QTL detection were determined at the experiment-wise error of *P* > 0.05 by computing 1000 permutations, using QTL CARTOGRAPHER. QTLs were determined when LOD scores were greater than 2.5.

### Statistical analysis

Two-way analysis of variance (ANOVA) was used to evaluate the effects of alleles, cold stress conditions and their interactions by comparing the CTS scores of RIL groups possessing respective alleles at SSR markers nearest to the detected QTLs after the first and second cold stress periods. In the ANOVA array, four means of CTS scores in four subclasses composed of two factors (alleles and stress conditions) were calculated using bioassay data obtained in three replicated experiments, each consisting of 4 × 5 seedlings.

## Results and discussion

### Bioassays for phenotyping CTG and CTS

**Figure 1.**
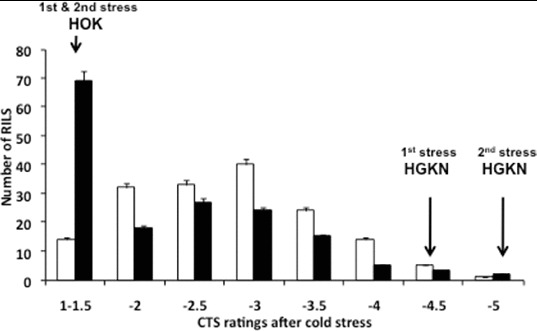
Frequency distribution of RILs in the five-point rating scales for CTS obtained after the first period of cold stress (white bars) and after the second period of cold stress with an intervening eight-day recovery period (black bars). Error bars indicate standard errors of the means of the eight rating classes. CTS data after the first period of cold stress are from [36]. For the five-point rating scales, see Materials and methods.

The two parental cultivars showed a clear difference in their rate of germination under the described cold stress condition. The *indica* parent HOK showed a very low germination rate ranging from 0% to 8.0% with an average of 2.6% in 10 repeated experiments. On the other hand, the *japonica* parent HGKN showed a much higher germination rate ranging from 66.7% to 91.6% with an average of 77.3%. Plotting the percentage germination rates of 162 RILs yielded a normal distribution, indicating that CTG was under polygenic control and that our bioassay conditions could serve as a useful means for detecting QTLs for CTG.[36] For evaluating CTS, cold stress was given in two consecutive periods of three days with an intervening recovery period of eight days. After the first period of cold stress, the majority of HOK seedlings withered during the five days of the recovery period with an average rating of 1.5 ranging from 1 to 2, while the majority of HGKN seedlings remained green with an average rating of 4.4 ranging from 3 to 5. RILs showed a normal distribution of the ratings with a little skewness towards cold susceptibility in the first period of cold stress (Figure 1).

We further subjected a whole set of RILs to a second period of cold stress after an intervening eight-day recovery period. The experiment was performed in order to examine the effect of the repeated and severer cold stress with an intervening recovery period at normal temperature on CTS. All HGKN seedlings survived with a rating score of 5 after the second period of cold stress, whereas all HOK seedlings withered and died at an average rating of less than 1.5 (Figure 1). The ANOVA test of the ratings after the second period of cold stress demonstrated a significant variability between RILs but no differences within RILs after the first period (data not shown), indicating that seedling response to the cold stress was homogeneous within each RIL during both cold stress periods. RILs after the second period of cold stress, however, showed a highly skewed distribution towards cold susceptibility. Seventy RILs (43.2%) withered and died at the second period of cold stress with average rating scores below 1.5. The result showed that the two repeated periods of cold stress with an intervening recovery period caused more damage to rice seedlings than that caused by the first period of cold stress.

**Figure 2.**
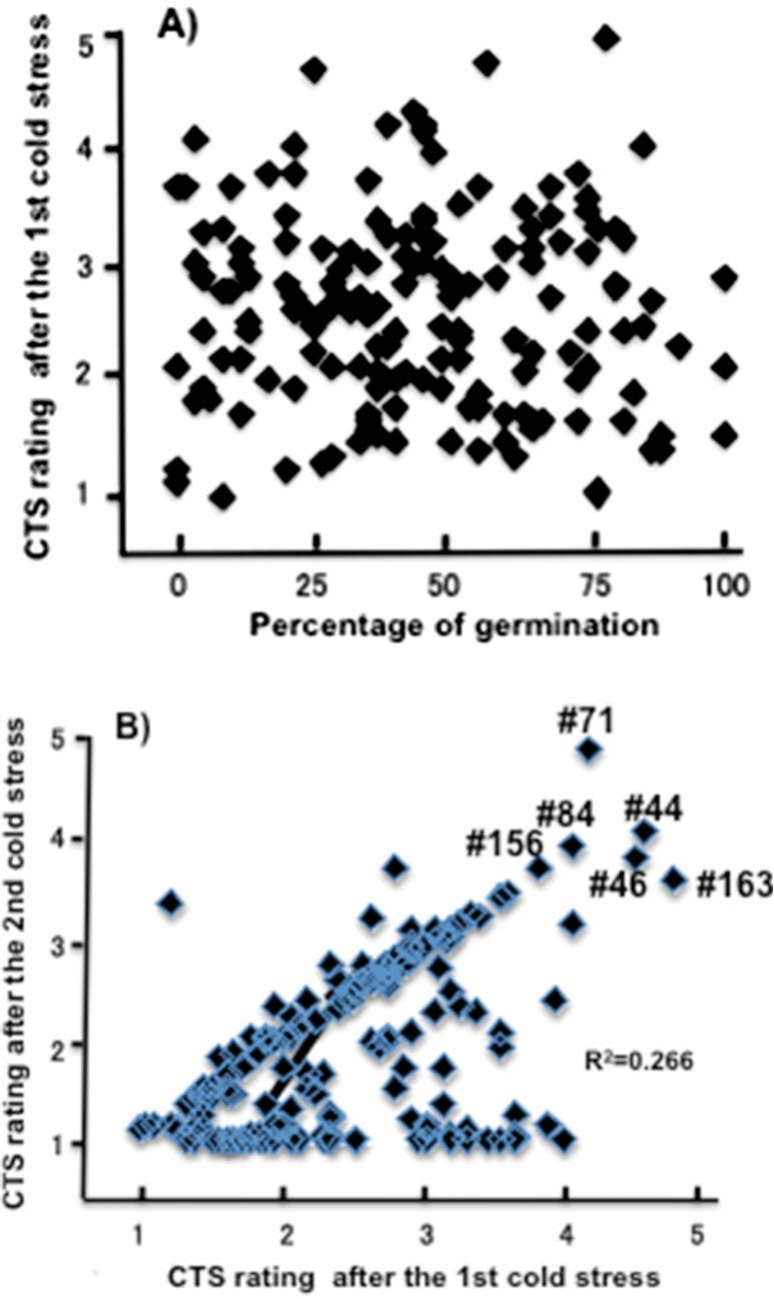
Correlations between the levels of CTG and CTS. (A) No correlations between CTG and CTS after the first period of cold stress. (B) A weak but significant correlation (*R* ^2^ = 0.266) between CTS after the first period of cold stress and that after the second period of cold stress. RILs #44, 46, 71, 84, 156 and 163 showed CTS scores higher than 3.5 after both stress periods.

No correlations were observed between CTG and CTS both after the first period of cold stress (*r* = 0.05362, *p* = 0.5007 in Figure 2(A)) as well as after the second period (*r* = −0.03097, *p* = 0.6974). On the other hand, a weak correlation (coefficient of determination *R* ^2^ = 0.266, *p* << 0.0001) was observed in CTS rating scores between the two cold stress periods (Figure 2(B)). Under our experimental conditions, the CTS rating scores after the second period of cold stress in fact contained effects of the whole experiment including those of both the first and the second stress period. The presence of a weak correlation might suggest the involvement of some unknown interactive factors that influenced the second CTS rating scores.

### Identification of QTLs for CTG and CTS and their developmental specificity

**Figure 3.**
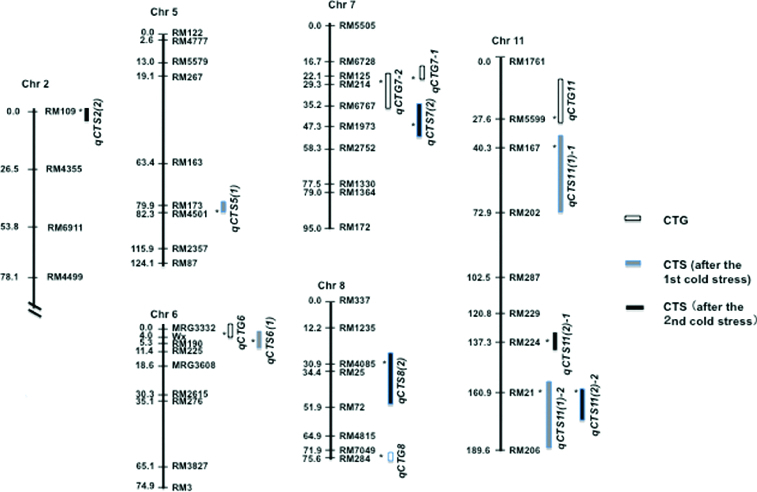
A linkage map constructed using 162 RILs and 102 SSR markers and positions of QTLs associated with cold tolerance at germination (*qCTG*) and seedling stage (*qCTS*). Positions of QTLs and the nearest markers were according to LOD plots obtained by QTL CARTOGRAPHER. For nomenclature of QTLs, see Table 1.

A framework linkage map was constructed, with an average marker density of 15.2 cM, spanning the 1552 cM total length of the 12 rice chromosomes. Combining the phenotypic data and the linkage map, composite interval mapping was performed to identify QTLs associated with CTG and CTS (Table 1 and Figure 3). For CTG, five QTLs were detected on chromosomes 6, 7, 8 and 11, among which *qCTG7-1* and *qCTG7-2* were overlapped between markers RM125 and RM214 on the short arm of chromosome 7. The total phenotypic variance (PVE) explained by the five QTLs ranged from 5.7% to 9.3%. At the three QTLs on chromosomes 6 and 7, alleles from the cold tolerant *japonica* parent HGKN gave negative additive effects. At the two other QTLs on chromosomes 8 and 11, on the other hand, HGKN alleles gave positive additive effects and thus contributed to increase the level of CTG.

**Table 1.** List of cold tolerance QTLs at germination (CTG) and the seedling stage (CTS) detected by composite interval mapping.

Trait/Locus	Marker interval	Peak marker (cM)^1^	LOD score	PVE^2^	a^3^
Germination stage					
*qCTG6*	MGR3332-Wx	Wx (4.1)	2.6	5.7	−6.7
*qCTG7-1*	RM6728-RM125	RM125 (18.2)	2.6	6.4	−6.6
*qCTG7-2*	RM125-RM1973	RM214 (28.3)	3.7	9.3	−8.8
*qCTG8*	RM7049-RM284	RM284 (75.2)	2.7	6.4	6.6
*qCTG11*	RM1761-RM167	RM5599 (17.7)	3.2	5.8	9.8
Seedling stage (after the first cold stress)					
*qCTS5(1)*	RM163-RM4501	RM4501 (82.0)	2.7	5.8	−0.24
*qCTS6(1)*	Wx-RM225	RM190 (6.3)	3.5	8.5	0.25
*qCTS11(1)-1*	RM167-RM202	RM167 (56.2)	3.4	22.2	0.36
*qCTS11(1)-2*	RM224-RM206	RM21 (165.7)	11.7	35.6	0.50
Seedling stage (after the second cold stress)					
*qCTS2(2)*	RM109-RM4355	RM109 (2.9)	2.8	22.9	0.24
*qCTS7(2)*	RM6767-RM2752	RM1973 (45.7)	6.4	35.3	0.34
*qCTS8(2)*	RM1235-RM72	RM4085 (34.5)	3.9	27.2	0.28
*qCTS11(2)-1*	RM229-RM21	RM224 (137.5)	3.4	6.5	−0.23
*qCTS11(2)-2*	RM21-RM206	RM21 (160.9)	6.7	12.5	0.44

^1^Peak position in cM from the end of the short arm.

^2^Percentage of total PVE explained by QTL.

^3^Additive effect of HGKN (*japonica*) allele.

For CTS after the first three-day period of cold stress, four QTLs were detected on three chromosomes 5, 6 and 11. *qCTS11(1)-1* located on the short arm of chromosome 11 and *qCTS11(1)-2* on the long arm of chromosome 11 showed 22.2% and 35.6% PVE, respectively, while the other two QTLs, *qCTS5(1)* on the long arm of chromosome 5 and *qCTS6(1)* on the short arm of chromosome 6, respectively, showed 5.8% and 8.5% PVE. *qCTS6(1)* was overlapped with *qCTG6* within the chromosomal region covered by markers Wx and RM190. Positive additive effects were conferred by HGKN alleles at all QTLs except for *qCTS5(1)*. For CTS after the second period of cold stress, five QTLs were detected on chromosomes 2, 7, 8 and 11. Among them, *qCTS2(2)*, *qCTS7(2)* and *qCTS8(2)* showed PVE ranging from 22.9% to 35.3%, while two other QTLs, *qCTS11(2)-1* and *qCTS11(2)-2*, showed 6.5% and 12.5% PVE, respectively. *qCTS11(2)-2* was overlapped with *qCTS11(1)-2* in the chromosomal region covered by markers RM21 and RM206. PVE of *qCTS11(2)-2* was lower than that of *qCTS11(1)-2*, although their additive effects were similar. The closest marker for these two QTLs was RM21. At all these QTLs except for *qCTS11(2)-1*, *japonica* HGKN alleles contributed to increase the level of CTS.

A large volume of research has been conducted to identify QTLs associated with cold tolerance at various developmental stages in rice. With respect to CTG and CTS, the presence of common QTLs was suggested in several studies using the same sets of mapping populations.[27,28,45,46] Chromosomal positions of *qLTG-11* [27] and *qSCT-11* [33] for CTS appeared to be overlapping. However, the regions covered by these QTLs were large, spanning across the centromere. Bertin et al. [47] reported a positive correlation of CTS at the two-leaf and eight-leaf stages. Similarly, Baruah et al. [31] reported that two QTLs located in a similar chromosomal region were commonly active at both the plumule stage (1.0–1.5 cm long plumule after germination) and the seedling stage (12-day-old). In our study, all but two QTLs, *qCTG6* and *qCTS6(1)*, were stage specific and contributed either only to CTG or to CTS. These two QTLs overlapping at markers Wx and RM190 on the short arm of chromosome 6, however, showed opposite effects on the levels of CTG and CTS. Furthermore, no significant correlations were observed between the levels of CTG and CTS (Figure 2(A)). Our results showed that all positive QTLs increasing the levels of CTG and CTS were developmental stage specific and active either at germination or at the early seedling stage in our mapping population.

### Effects of alleles, cold stress conditions and their interactions on CTS scores of RILs at QTLs

We compared the mean CTS scores of RIL groups possessing either HGKN alleles or HOK alleles at the SSR marker sites nearest to the QTLs associated with CTS after the first and second period of cold stress (Table 2). At all these QTL marker sites, RILs with HGKN alleles showed higher mean CTS scores than those with HOK alleles. Mean CTS scores after the first cold stress period were higher than those after the second cold stress period irrespective of the alleles at the QTL marker sites. We therefore conducted a two-way ANOVA test to compare the effects of alleles and stress conditions and also their interactions on mean CTS scores (Table 3). The test showed that the effects of alleles, stress conditions and their interactions were all highly significant at four QTL marker sites (RM21, 109, 1973 and 4085), while at four other sites (RM 4501, 190, 167 and 224) no significant interactions were observed, although allele effects were significant. We also studied the segregation pattern of the alleles at marker RM21, which was located at the nearest site of the two overlapping QTLs on chromosome 11, *qCTS11(1)-2* and *qCTS11(2)-2*, in RILs showing higher than 3.5 CTS rating scores for both after the two stress conditions. The study showed that five (#44, 46, 71, 84 and 156) of six such RILs (shown in Figure 2) possessed HGKN alleles in the homozygous condition. One exceptional RIL #163 possessed a HOK allele at this marker site, indicating that some other QTLs might have contributed to increase its level of CTS.

**Table 2.** Mean CTS scores of RILs possessing HGKN or HOK alleles at SSR markers nearest to QTLs associated with CTS after the first and second cold stress.

Marker	QTL	Allele	First stress	Second stress
RM4501	*qCTS5(1)*	HGKN	2.462 ± 0.002	2.390 ± 0.007
		HOK	2.330 ± 0.003	2.247 ± 0.008
RM190	*qCTS6(1)*	HGKN	2.766 ± 0.010	2.315 ± 0.007
		HOK	2.348 ± 0.000	1.753 ± 0.000
RM167	*qCTS11(1)-1*	HGKN	2.651 ± 0.001	2.146 ± 0.004
		HOK	2.294 ± 0.005	1.846 ± 0.000
RM21	*qCTS11(1)-2*, *11(2)-2*	HGKN	2.762 ± 0.001	2.327 ± 0.007
		HOK	2.332 ± 0.005	1.626 ± 0.001
RM109	*qCTS2(2)*	HGKN	2.676 ± 0.000	2.188 ± 0.001
		HOK	2.440 ± 0.003	1.748 ± 0.001
RM1973	*qCTS7(2)*	HGKN	2.663 ± 0.001	2.327 ± 0.004
		HOK	2.346 ± 0.004	1.622 ± 0.001
RM4085	*qCTS8(2)*	HGKN	2.538 ± 0.007	2.148 ± 0.002
		HOK	2.400 ± 0.001	1.643 ± 0.002
RM224	*qCTS11(2)-1*	HGKN	2.576 ± 0.004	2.065 ± 0.007
		HOK	2.448 ± 0.003	1.862 ± 0.000

Note: Mean CTS scores were calculated as grand means of three replicated experiments each consisting of 4 × 5 seedlings. Figures after ± represent standard errors.

**Table 3.** Two-way ANOVA for evaluating interaction between alleles and cold stress conditions at SSR markers nearest to QTLs associated with CTS after the first and second cold stress.

Marker	QTL	Factor	MS	*F* value
RM4501	*qCTS5(1)*	Allele	0.0565	11.264**
RM190	*qCTS6(1)*	Allele	0.7215	167.047***
		Stress	0.8209	190.085***
RM167	*qCTS11(1)-1*	Allele	0.3234	118.450***
		Stress	0.6813	249.546***
RM21	*qCTS11(1)-2*, *11(2)-2*	Allele	0.9594	284.279***
		Stress	0.9765	289.359***
		Interaction	0.0545	16.163**
RM109	*qCTS2(2)*	Allele	0.3426	232.914***
		Stress	1.0453	710.578***
		Interaction	0.0314	21.321**
RM1973	*qCTS7(2)*	Allele	0.7853	341.888***
		Stress	0.8429	366.936***
		Interaction	0.1129	49.149***
RM4085	*qCTS8(2)*	Allele	0.3107	104.844***
		Stress	0.9863	332.813***
		Interaction	0.1012	34.139***
RM224	*qCTS11(2)-1*	Allele	0.0821	23.131**
		Stress	0.9034	254.623***

Note: Only significant factors (alleles and stress conditions) and their interactions are shown. *qCTS11(1)-2* and *qCTS11(2)-2* were overlapped at the marker RM21. ** and *** represent significance at the 1.0% and less than 0.1% level, respectively.

### Comparison of QTLs identified so far

Comparison of chromosomal locations of the QTLs with those of previously reported ones might provide us with some information on their differences and/or similarities, although there is a limitation due to different marker systems and materials used. We thus surveyed the past research results reported on QTLs for CTG and CTS. By comparing QTLs identified in this study (Table 1 and Figure 3) and ones previously reported, the following inferences could be drawn. Three QTLs for CTG, *qCTG6*, *8* and *11*, three for CTS after the first cold stress period, *qCTS5(1)*, *6(1)* and *11(1)-1*, and one for CTS after the second stress period, *qCTS2(2)*, appeared to be novel. Among the nine QTLs for CTS detected after the first and second period of cold stress, *qCTS7(2)* was overlapped with *qSES7-1* and *qSES7-2*.[48] *qCTS8(2)* was located in a similar chromosomal region to that of *qCTS8.1*.[46] The positions of the two overlapping QTLs, *qCTS11(1)-2* and *qCTS11(2)-2*, were within a chromosomal region of *Chr11*, which was first identified by Misawa et al. [35] on the long arm of rice chromosome 11, using F_2-3_ lines derived from the original *japonica* × *indica* cross. Furthermore, previously reported QTLs such as *qCTS-11* for seedling vigour,[33] *qCTS11* and *qCTP11* for seedling growth and plumule growth,[31] respectively, and *qCTS11-2* for cold-induced necrosis tolerance at the seedling stage [25] were all located in a similar region on chromosome 11. The short arm of chromosome 11 also possessed *qCTS11-1* for cold-induced wilting tolerance [25] and *qSCT-11* for recovery growth after cold stress.[33] These results suggest that chromosome 11 possesses several major QTLs controlling cold tolerance at germination and the early seedling stages in rice. It has been well known that rice chromosome 11 has synteny with chromosome 5A of wheat and 5H of barley,[49] which possess the cluster of *FR2* (Frost Resistance 2) and *CBF* genes [50,51]. A detailed QTL analysis using more closely linked markers and a more powerful substitution mapping is required for further dissection of these QTL regions.

### Possible involvement of cold acclimation

It has been pointed out that differences in methods of cold treatment, including severity and duration of exposure to low temperature, can affect QTL detection in different experiments using the same mapping populations.[21] For example, Zhang et al. [33] reported that the effect of *qSCT-11* for recovery growth and seedling cold tolerance increased as the duration of cold stress was prolonged from 10 to 13 days. We attempted to study possible effects on CTS of the second cold stress given after the first cold stress with an intervening eight-day recovery period. We expected to detect robust QTLs effective under this more severe stress condition. We also expected to detect a different set of QTLs that could be activated during the first period of cold stress and became effective in the second stress period. In fact, we observed an increase in the CTS rating of HGKN after the second stress period compared with that after the first period (Figure 1). We also detected four different QTLs after the second period of cold stress (Table 1). Among them, *qCTS7(2)* with PVE of 35.3% (Table 1) was likely identical to the one we detected previously using the same set of RILs but under different bioassay conditions.[52] The detection of different QTLs after the two different stress conditions indicated possible interactions between QTLs and cold stress conditions. We therefore conducted a two-way ANOVA test and found significant interactions between allele types at the QTL sites and the two stress conditions with respect to the mean CTS scores (Table 3).

It is known that the cold-responsive signal pathway, which is dependent on *CBF/DREB1* (C-repeat binding factor/dehydration responsive-element binding protein 1), plays a key role in cold acclimation in plants. The molecular basis of cold acclimation and resulting acquired freezing tolerance in *Arabidopsis* and in winter cereals, including wheat and barley, is well documented.[12,51,53–56] By contrast, the presence of a similar cold acclimation network remains controversial in highly cold-sensitive rice of tropical origin. The rice genome, however, has recently been shown to possess a number of putative *CBF/DREB1* orthologues, some of which were suggested to be involved in cold acclimation.[57,58] With respect to root water uptake, the presence and function of a cold acclimation process has been shown to be under regulation by root aquaporins in rice.[59] Our results showing the strong interaction between QTLs and cold stress conditions suggest the possible involvement of cold acclimation process in rice for CTS. Optimization of bioassay conditions is needed in order to confirm this intriguing possibility.

### Implications in practical rice breeding programs for cold tolerance

*O. sativa* ssp. *japonica* is confined mostly to the temperate regions, while *indica* ssp. is grown in the tropical regions.[60,61] Thus, *indica* generally possesses much lower levels of cold tolerance than *japonica* throughout the developmental stages of rice. Cold stress often associated with submergence is a serious threat for *indica* rice particularly in the regions where direct seeding cultivation system is practiced under low local temperatures in Asia.[9–11] We showed that *japonica* alleles contributed to enhance the level of cold tolerance at a majority of QTLs detected (Table 1). The result strongly suggests the potential usefulness of *japonica* germplasms in improving cold tolerance in *indica* rice, which provides a large population in Asia with a source of staple diet. Further studies focused on major QTLs detected on chromosomes 2, 7, 8 and 11 (Table 1 and Table 2) are needed to explore this promising possibility.

## Conclusions

We detected several major QTLs associated with cold tolerance at germination and the early seedling stage in rice, using RILs obtained from a *japonica* × *indica* cross. At a majority of QTLs *japonica* alleles contributed to enhance cold tolerance, explaining 6.4%–35.6% of the total PVE. All but one QTL were effective either to CTG or CTS, indicating that cold tolerance QTLs were mainly growth-stage specific. Not only the same but also different QTLs were detected for CTS after the two consecutive periods of cold stress with an intervening recovery period, indicating a possible involvement of cold acclimation process in rice seedlings. Our results also suggested a promising role of *japonica* germplasms in breeding for cold tolerance in *indica* rice.
